# Plasma amino acid concentrations at admission and 28-day mortality in ST-elevation myocardial infarction

**DOI:** 10.1186/s12986-026-01139-8

**Published:** 2026-05-20

**Authors:** Christa Meisinger, Dennis Freuer, Philip Raake, Jakob Linseisen, Timo Schmitz

**Affiliations:** 1 Epidemiology, Medical Faculty, University of Augsburg, Stenglinstraße 2, 86156 Augsburg, Germany; 2 Department of Cardiology, Respiratory Medicine and Intensive Care, University Hospital Augsburg, 86156 Augsburg, Germany

**Keywords:** Amino acids, Short-term mortality, Acute myocardial infarction

## Abstract

**Background:**

Amino acid metabolism plays a critical role in cardiovascular disease, yet its prognostic value in ST-elevation acute myocardial infarction (STEMI) remains underexplored. Therefore, we investigated whether specific plasma amino acid concentrations at admission for STEMI are associated with 28-day mortality.

**Methods:**

This analysis was based on data from 724 patients with STEMI aged 29 to 98 years who were admitted to the University Hospital Augsburg between May 2009 and July 2013. Immediately after admission arterial blood samples were taken from these patients and a panel of amino acids was measured by a high-throughput nuclear magnetic resonance spectroscopy platform (Nightingale Health, Finland). Multivariable logistic regression models were conducted to examine the associations between the amino acids phenylalanine, tyrosine, glycine, alanine, histidine, glutamine, as well as branched-chain amino acids (BCAAs; a group that includes valine, isoleucine, and leucine) and 28-day mortality. P values were False discovery rate (FDR) adjusted.

**Results:**

Altogether, 47 patients died within 28 days after admission. There were significant positive associations found between plasma levels of phenylalanine, glycine, tyrosine, valine, and alanine and 28-day mortality. Phenylalanine showed the highest effect estimate (OR: 1.84; 95% CI 1.34–2.53). No significant associations were observed for the remaining amino acids.

**Conclusions:**

The acute phase of STEMI is associated with changes in plasma amino acid levels that may reflect alterations in energy metabolism and metabolic stress. The associations between amino acid fluctuations and 28-day mortality highlight the potential of metabolomic profiling to refine early risk stratification.

## Background

Cardiovascular disease remains the leading cause of mortality worldwide, with atherosclerosis representing the predominant underlying pathological process [1]. The global prevalence of coronary atherosclerosis has increased steadily over recent decades, contributing to a corresponding rise in deaths attributable to ST-elevation acute myocardial infarction (STEMI) [2]. STEMI results from sudden coronary occlusion and is characterized by acute ischemic injury, cardiomyocyte necrosis, and a complex systemic response involving inflammation, neurohormonal activation, and metabolic dysregulation [3]. Current clinical risk stratification in acute myocardial infarction primarily relies on demographic characteristics, cardiac biomarkers, electrocardiographic findings, and hemodynamic parameters [4]. While these measures are essential for diagnosis and management, they do not fully capture the biological diversity that influences disease severity, early complications, and recovery after infarction. Therefore, there is a need for novel approaches that can assess the integrated molecular and metabolic responses to acute ischemic stress [5].

Metabolomics, which involves the comprehensive profiling of low-molecular-weight metabolites, offers a powerful approach to investigate systemic metabolic alterations in STEMI. By reflecting the downstream effects of genetic, environmental, and pathophysiological factors, metabolomic profiles can provide insights into disease mechanisms and help identify novel diagnostic and prognostic biomarkers [6]. In the context of acute myocardial infarction, metabolomics enable the detection of acute metabolic responses that may not be captured by conventional clinical markers [7].

In addition to disturbances in lipid and glucose metabolism, growing evidence highlights the important role of amino acid metabolism in cardiovascular disease [2]. Amino acids regulate energy production, redox balance, inflammation, and cellular signaling [8]. These processes are central to myocardial ischemia, injury, and post-infarction remodeling [9]. Altered levels of specific amino acids, such as phenylalanine, tyrosine, glycine, and alanine, as well as the branched-chain amino acids (BCAAs; a group that includes valine, isoleucine, and leucine), have been linked to cardiovascular pathophysiology and adverse clinical outcomes [10, 11].

Collectively, these observations suggest that dysregulated amino acid metabolism may reflect aspects of the systemic response to STEMI and could provide prognostic information beyond traditional risk markers. Therefore, investigating amino acid profiles in STEMI patients is a promising approach for improving risk stratification and understanding disease mechanisms. To address this gap, we investigated whether specific plasma amino acid concentrations at admission for STEMI are associated with 28-day mortality.

## Methods

### Study population

This study used data from the population-based Augsburg Myocardial Infarction Registry in Germany. The registry has been operating since 1984, when it was established as part of the MONICA project (Monitoring Trends and Determinants in Cardiovascular Disease). From 1996 to 2020, it was conducted as the KORA (Cooperative Health Research in the Augsburg Region) Myocardial Infarction Registry, and since 2021 it has continued as the Augsburg Myocardial Infarction Registry at the University Hospital of Augsburg. The study region includes approximately 705,000 inhabitants, covering the city of Augsburg and the surrounding counties of Augsburg and Aichach-Friedberg. All patients with acute myocardial infarction admitted consecutively to one of the seven hospitals in the study area were registered, provided they were aged 25–74 years (extended to 25–84 years since 2009) and had their primary residence in the study region at the time of the event. Detailed information on case identification, diagnostic classification, and data quality control has been published previously [12, 13].

For the present analysis, patients with ST-elevation myocardial infarction (STEMI) admitted to the University Hospital of Augsburg between May 2009 and July 2013 were included. Only patients who were transferred to the cardiac catheterization laboratory for interventional therapy following hospital admission were considered. Of those, only patients for whom blood samples were available were included in the analysis. Patients who did not consent to take part in the study were not included.

Blood samples were collected during cardiac catheterization, which was usually performed immediately after hospital admission and stored at − 80 °C until analysis. In 2023, a panel of nuclear magnetic resonance (NMR) metabolic biomarkers generated by Nightingale Health was measured. Of the initial 793 patients, 67 were excluded due to missing valid concentrations for all measured amino acids, resulting in 724 cases for statistical analysis. An additional 4 patients lacked information on diabetes or renal function and were therefore excluded from the regression analysis.

The study (original data collection) was approved by the ethics committee of the Bavarian Medical Association (Bayerische Landesärztekammer), approval number 12,057. Furthermore, the collection of blood was approved by the ethics committee of the Bavarian Medical Association, approval number 09016. The study and blood collection were performed in accordance with the Declaration of Helsinki.

### Data collection

All patients with acute myocardial infarction were interviewed during their hospital stay by trained study nurses using a standardized questionnaire. In addition, patients’ medical records were reviewed to obtain comprehensive information, including demographic characteristics, cardiovascular risk factors, medical history, comorbidities, medication use, laboratory parameters, and electrocardiographic findings.

Blood samples from the patients were collected during cardiac catheterization, which generally was performed immediately after hospital admission. Arterial EDTA blood samples were obtained at the beginning of the catheterization procedure and immediately processed in a standardized manner in the catheterization laboratory, including centrifugation, aliquoting, and storage at − 80 °C.

### Clinical chemistry measurement

Routine blood parameters were measured in venous blood samples taken at hospital admission or during hospital stay as part of the regular diagnosis and treatment.

Plasma concentrations of amino acids (phenylalanine, glycine, valine, alanine, tyrosine, histidine, isoleucine, leucine, glutamine, and total branched-chain amino acids) were measured by a high-throughput nuclear NMR spectroscopy platform (Nightingale Health, Finland) [14, 15]. Measurements below the limit of quantification were included in the analysis if an extrapolated value was provided by Nightingale.

### Statistical analysis

Categorical variables were presented as absolute frequencies and percentages; Chi-square tests were applied to test for group differences. The variable age was displayed as mean and standard deviation (SD), all other continuous variables were presented as median and inter-quartile range (IQR). For comparison of group differences, Student’s t tests (for the variable age) and Mann–Whitney U tests (for other continuous variables) were used.

#### Logistic regression analysis

To ensure comparability between different amino acids, the plasma values for each amino acid were standardized (the variables were centered and normalized so that the transformed variable had an expectancy value of 0 and a statistical variance of 1). Values deviating more than 3 SD from the mean were excluded for regression analyses. Likewise, influential observations with a Cook´s d ≥ 0.5 were removed from further analyses. To examine the association between amino acids (exposures) and 28-day mortality (outcome), binary logistic regression models were calculated. Based on literature research, the models were adjusted for sex (male/female), age (in years), diabetes mellitus (yes/no), smoking status (current/former/never) body mass index (kg/m²), peak creatine kinase MB (CKMB) levels (U/L) and renal function (normal renal function [eGFR ≥ 60 ml/min/1,73 m²], slightly impaired renal function [eGFR 30-59.9 ml/min/1,73 m²], heavily impaired renal function [eGFR < 30 ml/min/1,73 m²]). Due to a right-skewed distribution, peak CKMB levels were square rooted for the regression analyses (not so for the baseline characteristics, Table 1), which resulted in a distribution close to normal distribution. Existing literature suggests that the association between BMI and mortality after STEMI is U-shaped [16], consequently we used restricted cubic splines with 4 knots (at the 5th, 35th, 65th and 95th percentiles) for the continuous BMI variable to account for potential nonlinearity. Since there was a substantial number of missing values for the variables BMI, smoking and peak CKMB (see Table 1), multivariate Imputation by Chained Equations was performed using R´s mice package [17]. The number of imputed data sets was to 5. All 5 imputed data sets were stacked to a combined data frame. The logistic regression models were then calculated on the stacked data set using weights of 0.2 for each observation. Cases with missing values on diabetes and renal function were removed from the regression analyses. False discovery rate (FDR) adjustment of the obtained *p*-values was conducted to control for multiple testing. The effect estimates (odds ratio and 95% CI) from the logistic regression models must be interpreted as the expected increase of 28-day mortality chance with every increase of one standard deviation in the respective exposure variable (plasma amino acid concentration).

**Table 1:** Baseline characteristics of the STEMI patients for the total sample and stratified by 28-day mortality

	*Total sample (* *N* * = 724)*	*28-days survived (* *N* * = 677)*	*Died within 28-days (* *N* * = 47)*	*P*-Value	*N*
**Age**	63.4 (12.6)	62.8 (12.5)	73.1 (10.2)	< 0.001	724
Sex (male)	545 (75.3)	518 (76.5)	27 (57.4)	0.006	724
Reinfarction (yes)	70 (9.7)	65 (9.6)	5 (10.6)	1	723
Typical chest pain (yes)	622 (86.9)	598 (88.6)	24 (58.5)	< 0.001	716
Prehospital delay (min)	119.0 (70.0–281.0)	120.0 (72.5–286.5)	87.0 (51.8–176.8)	0.025	653
LVEF < 30%	56 (8.0)	42 (6.4)	14 (35.0)	< 0.001	697
Peak CKMB (U/L)	155.0 (68.2–294.0)	152.5 (67.8–279.2)	223.0 (95.8–408.2)	0.041	694
**Renal Function**				< 0.001	724
eGFR normal	494 (68.5)	477 (70.7)	17 (37.0)		721
eGFR slightly impaired	194 (26.9)	173 (25.6)	21 (45.7)		
eGFR severly impaired	33 (4.6)	25 (3.7)	8 (17.4)		
Diabetes mellitus (yes)	198 (27.4)	184 (27.2)	14 (29.8)	0.832	723
Hypertension (yes)	519 (71.9)	483 (71.4)	36 (78.3)	0.409	722
Hyperlipidemia (yes)	360 (49.9)	343 (50.7)	17 (37.0)	0.098	722
**Smoking**				0.333	662
Current smoker	289 (43.7)	275 (43.1)	14 (58.3)		
Former smoker	178 (26.9)	173 (27.1)	5 (20.8)		
Never smoker	195 (29.5)	190 (29.8)	5 (20.8)		
Body mass index (kg/m²)	27.0 (24.7–30.1)	27.0 (24.7–30.1)	25.8 (24.2–30.1)	0.27	690
PTCA (yes)	658 (91.0)	621 (91.9)	37 (78.7)	0.005	723
Bypass therapy (yes)	67 (9.3)	57 (8.4)	10 (21.3)	0.007	723
**Amino Acids (mmol/l)**					
Phenylalanine	0.07 (0.06–0.08)	0.07 (0.06–0.08)	0.08 (0.07–0.12)	< 0.001	713
Glycine	0.21 (0.18–0.24)	0.20 (0.18–0.24)	0.24 (0.20–0.29)	0.001	705
Valine	0.22 (0.20–0.26)	0.22 (0.20–0.26)	0.24 (0.20–0.28)	0.052	723
Alanine	0.30 (0.25–0.36)	0.30 (0.25–0.36)	0.35 (0.27–0.48)	0.002	724
Tyrosine	0.06 (0.05–0.07)	0.06 (0.05–0.07)	0.06 (0.06–0.08)	< 0.001	721
BCAA (total)	0.40 (0.34–0.46)	0.40 (0.34–0.46)	0.44 (0.37–0.52)	0.044	723
Histidine	0.07 (0.06–0.08)	0.07 (0.06–0.08)	0.07 (0.06–0.09)	0.043	719
Isoleucine	0.06 (0.04–0.07)	0.06 (0.04–0.07)	0.06 (0.05–0.08)	0.298	724
Leucine	0.12 (0.10–0.14)	0.12 (0.10–0.14)	0.14 (0.11–0.17)	0.021	724
Glutamine	0.58 (0.52–0.65)	0.58 (0.52–0.65)	0.64 (0.52–0.75)	0.012	724

LVEF: left ventricular ejection fraction; CKMB: creatine kinase MB; eGFR: estimated glomerular filtration rate; PTCA: percutaneous transluminal coronary angioplasty;

BCAA: branched-chain amino acids

#### Receiver operating characteristic (ROC) analysis

ROC analyses were conducted to further evaluate the predictive ability of admission amino acids. Therefore, a logistic regression was calculated including all amino acids that were significantly associated with 28-day mortality in the regression analyses described above. A ROC analysis was then carried out using the linear predictor as the score value. To further contextualize we also performed a ROC analysis using the common epidemiological predictors sex and age. Finally, we combined the significant amino acids and sex/age to a common score and performed a final ROC analysis. In every ROC analysis, the 95% confidence interval (CI) for the area under the curve (AUC) was calculated using bootstrapping.

All statistical analyses were carried out with the statistical program R version 4.4.3. P values < 0.05 were regarded as statistically significant.

## Results

Of the 724 patients in the final study sample, 677 survived the first 28 days after hospitalization for STEMI, while 47 died within this period (see Table 1). The mean age of all included patients was 63.4 years; patients who survived the first 28 days were approximately 10 years younger than those who died. Three quarters of STEMI patients were men, but there was a significantly higher proportion of women among those who died. The two groups (survived/died within 28 days) also differed significantly in several characteristics, including left ventricular ejection fraction, peak CKMB levels, comorbidities, renal function, and acute therapy. For all analyzed plasma amino acids, levels were higher in patients who died within 28 days compared to those who survived, although not all differences reached statistical significance (see Table 1).

**Fig. 1:**
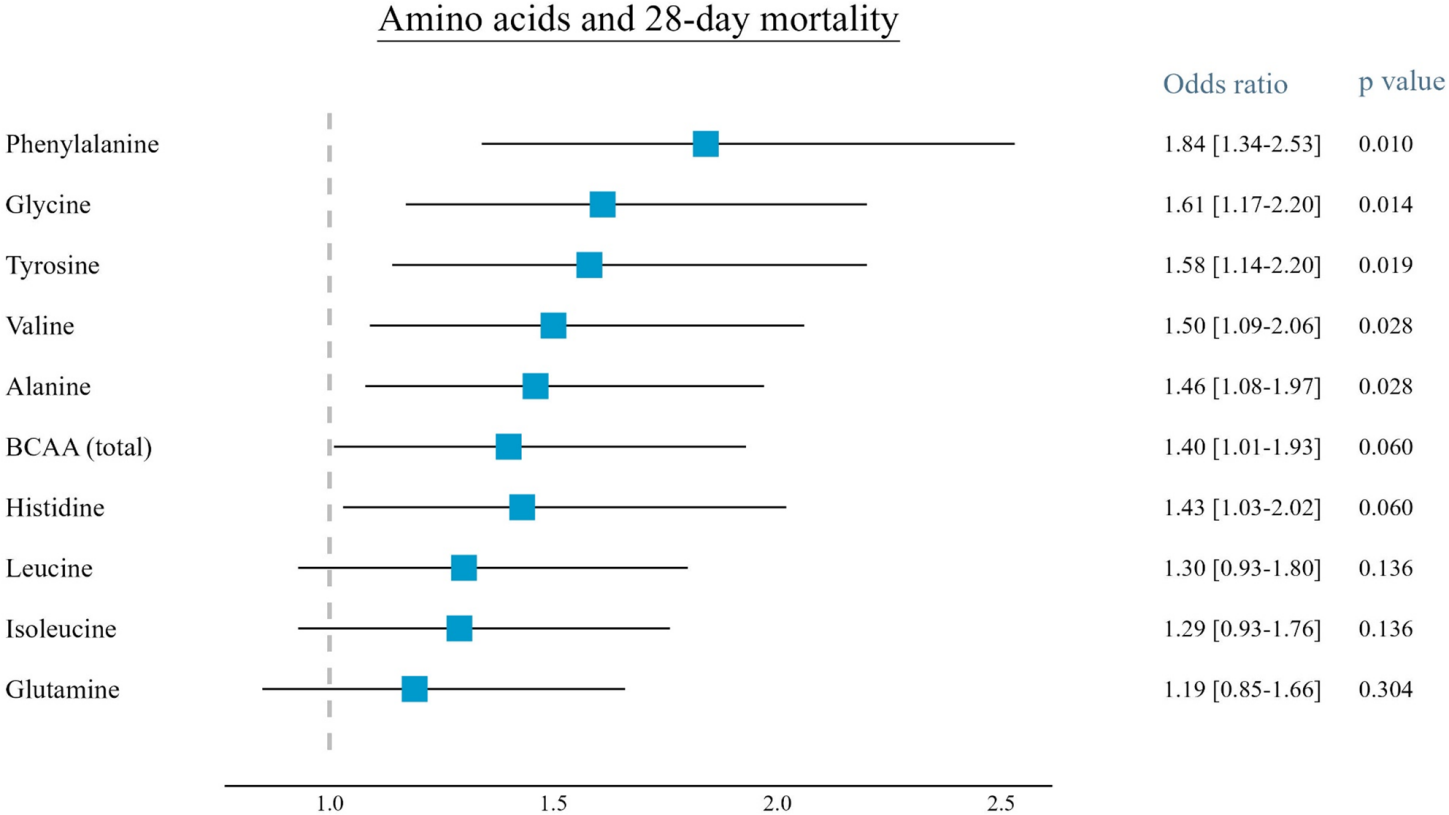
Results of the logistic regression models analyzing the association between admission plasma levels of amino acids and 28-day mortality in STEMI patients. Estimates are expressed as odds ratios and 95% confidence intervals. Presented p values are FDR-adjusted. BCAA: branched-chain amino acids

Regression analysis revealed significant positive associations between plasma levels of phenylalanine, glycine, tyrosine, valine, and alanine and 28-day mortality (see Fig. 1). Phenylalanine showed the highest effect size (OR: 1.84; 95% CI 1.34–2.53). Although increased odds ratios were observed for total BCAAs, histidine, isoleucine, leucine, and glutamine, these associations with 28-daymortality did not reach statistical significance.

**Fig. 2:**
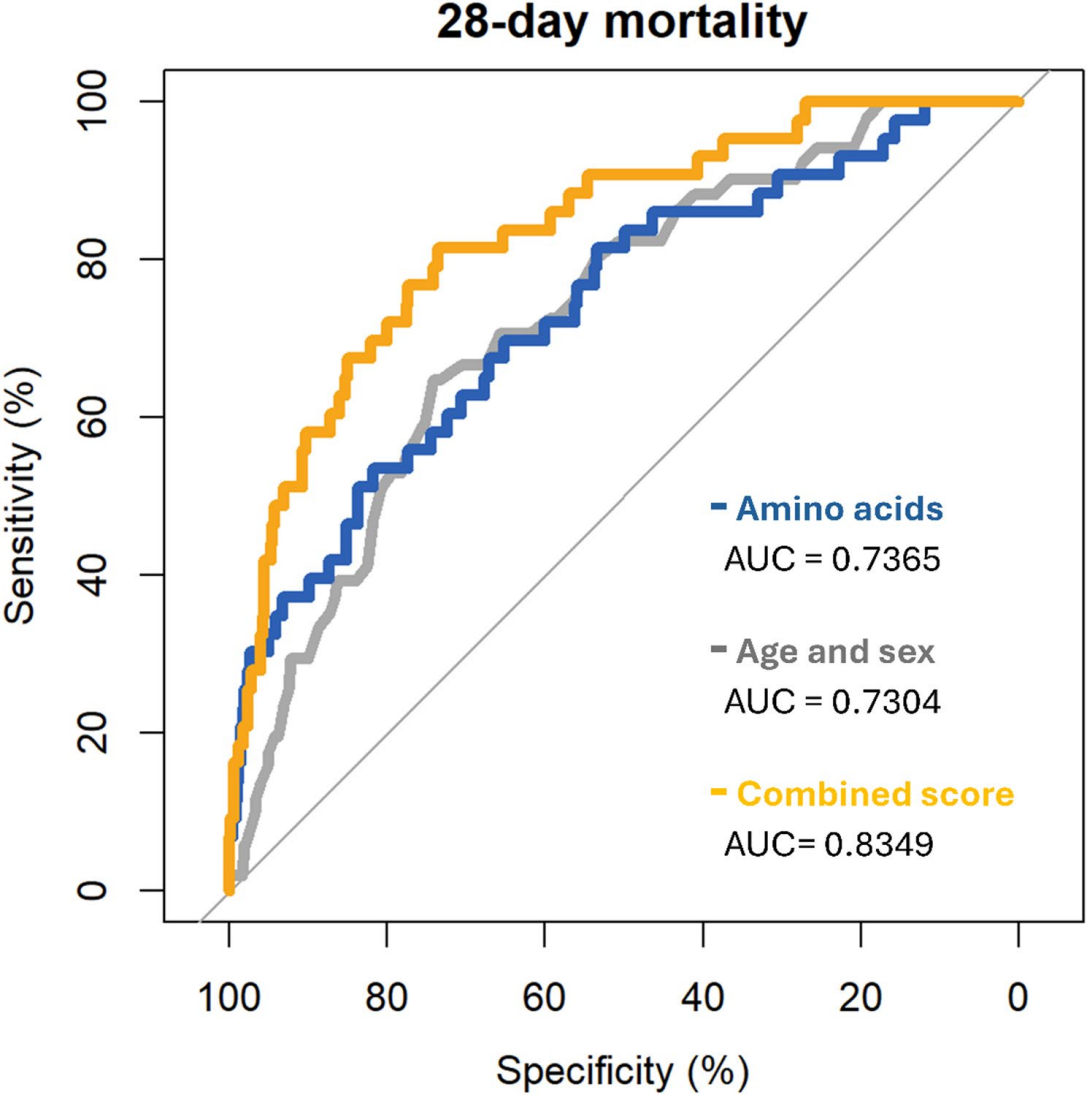
Results of the ROC analysis displaying the predictive ability of the five significantly associated amino acids (phenylalanine, glycine, tyrosine, valine, alanine), as well as sex and age and the combination of both to a combined score

The five significantly associated amino acids combined into a score showed decent predictive ability in a ROC analysis with an AUC of 0.7365 [95% CI 0.6542–0.8137], see Fig. 2. The inclusion of age and sex showed an AUC = 0.7304 [95% CI 0.6623–0.7955], and the combination of amino acids plus sex and age into a score resulted in a significantly better predictive power with an AUC of 0.8349 [95% CI 0.7708–0.8946].

## Discussion

In this population-based myocardial infarction registry study, we found that admission plasma levels of phenylalanine, tyrosine, glycine, valine, and alanine were positively associated with 28-day mortality in patients hospitalized with STEMI. The combination of these amino acids to a score underlined the strong predictive power regarding 28-day mortality.

The observed association between admission levels of phenylalanine and tyrosine and 28-day mortality reflects a profound metabolic shift triggered by STEMI. Both are aromatic amino acids that play interconnected roles in catecholamine synthesis and inflammatory regulation [18]. Increased circulating phenylalanine concentrations have been linked to reduced phenylalanine hydroxylase activity, a disturbance that may arise from oxidative stress and immune activation, both central mechanisms in the development of acute myocardial infarction [19, 20]. This enzymatic blockade, coupled with accelerated protein breakdown (proteolysis) in response to myocardial injury, leads to a rapid accumulation of phenylalanine [21]. Our findings align with previous research suggesting that this “stress hyperphenylalaninemia” is not merely a byproduct but strongly associated with 30-day mortality, reflecting the severity of the initial physiological insult [22].

Extending this metabolic perspective, previous studies have reported that lower admission levels of tyrosine are associated with poorer clinical outcomes, possibly reflecting a state of metabolic exhaustion or underlying malnutrition [23]. Tyrosine, as a fundamental precursor for the catecholamines dopamine, norepinephrine, and epinephrine, is essential for the neurohormonal stress response triggered by myocardial ischemia [24]. The discrepancy between our finding of elevated tyrosine levels being associated with increased mortality and these previous reports may be due to differences between chronic metabolic reserve and acute stress-induced tyrosine accumulation during severe ischemia. Additionally, disruptions in the metabolism of phenylalanine and tyrosine may indicate broader changes in neuroendocrine signaling and endothelial function, both of which are strongly associated with unfavorable cardiovascular outcomes in acute infarction [25, 26].

Expanding the metabolic scope, current evidence regarding the role of glycine as a marker for short-term complications in patients with STEMI is limited. In a study by Laremenko et al., glycine levels above 2.6 mg/dL were associated with persistent acute heart failure in 92 STEMI patients, demonstrating significant predictive capacity (OR 2.5, *p* < 0.0001) [27]. That research suggested that glycine could serve as a risk marker, particularly in patients with preserved left ventricular ejection fraction. Mechanistically, glycine is a conditionally essential amino acid (meaning it is usually synthesized by the body but may need to be obtained from the diet under certain conditions) recognized for its cytoprotective, anti-inflammatory, and antioxidative effects [28]. It regulates immune cell activity, suppresses excessive pro-inflammatory cytokine release, and contributes to the synthesis of glutathione, a major intracellular antioxidant [29, 30]. In prior studies, lower circulating glycine levels have been associated with increased oxidative stress and impaired endothelial function, both of which can worsen myocardial damage and hinder post-infarction repair processes [31, 32]. However, in our cohort, elevated circulating glycine levels during STEMI were associated with increased 28-day mortality. This paradoxical finding likely reflects severe hypercatabolic stress, organ dysfunction, and failure of compensatory protective mechanisms in critically ill patients, rather than a direct detrimental effect of glycine itself.

Likewise, the changes in plasma levels of the non-essential amino acid alanine observed at admission likely reflect a systemic shift in substrate metabolism in response to acute myocardial ischemia. In the early phase of STEMI, the abrupt transition to anaerobic metabolism and increased neurohormonal stress result in greater peripheral protein breakdown [33]. From a biological perspective, alanine acts as a primary transporter of amino groups from muscle and heart tissue to the liver, where it serves as a precursor for gluconeogenesis (the production of glucose from non-carbohydrate sources) [34]. This process, known as the glucose-alanine cycle, is essential for maintaining systemic glucose availability during periods of extreme physiological stress [35, 36]. During acute myocardial infarction, increased catabolic demand and insulin resistance can disrupt the balance (homeostasis) of alanine in the body, reflecting changes in systemic energy use [37]. Elevated circulating alanine levels may therefore serve as markers of metabolic stress, altered gluconeogenesis, and the overall physiological response to myocardial ischemia.

Shifting focus on valine, a branched-chain amino acid (BCAA), which plays a role in mitochondrial energy production and intracellular metabolic signaling. Impaired breakdown (catabolism) of BCAAs has been linked to cardiac insulin resistance, mitochondrial dysfunction, and maladaptive cardiac remodeling [38, 39]. Accordingly, the increase in circulating valine levels at admission in patients who died within 28 days, may paradoxically indicate impaired utilization of valine within cells and reduced adaptability of mitochondria to changing energy at the tissue level [40].

In this context, in the present study, apart from valine, no significant associations were found between other individual BCAAs or total BCAA levels and 28-day mortality. This finding contrasts with some previous studies in STEMI patients. For example, Du et al. reported that high BCAA concentrations at admission were associated with increased risks of in-hospital cardiovascular mortality and acute heart failure in 192 STEMI patients [41]. Another study demonstrated that BCAA levels were independent predictors of adverse cardiovascular events in 138 STEMI patients with acute heart failure (adjusted HR: 2.67, *p* < 0.001), with prognostic value surpassing that of established markers such as N-terminal pro-B-type natriuretic peptide [42]. However, these previous findings were based on relatively small cohorts, whereas our study included more than 700 STEMI patients. Further validation of our results in larger, independent cohorts is warranted.

### The metabolic signature of acute ischemic stress

The simultaneous changes in admission levels of phenylalanine, alanine, and valine highlight a complex systemic metabolic adaptation triggered by STEMI. Elevation of phenylalanine indicates impaired hydroxylation and increased systemic inflammation, while alterations in alanine and valine reflect a critical shift in energy substrate utilization [34]. Specifically, the mobilization of alanine via the glucose-alanine cycle and the increased use of the BCAA valine suggest a compensatory metabolic response to maintain energy balance through gluconeogenesis and alternative mitochondrial energy sources [43].

In the acute setting of a STEMI, these amino acid fluctuations may reflect changes in metabolic status beyond simple markers of nutritional status; they represent the degree of metabolic “decoupling” and mitochondrial strain [44]. Our findings suggest that a signature characterized by high stress-related amino acids (phenylalanine) and impaired utilization of energy substrates (valine) is associated with increased 28-day mortality. These metabolic changes may be associated with increased 28-day mortality. Systematic investigation of phenylalanine, tyrosine, glycine, alanine, and valine in STEMI patients may provide valuable insights into their association with short-term outcomes after myocardial infarction. This knowledge could inform future research aimed at understanding metabolic responses and developing novel prognostic markers in acute myocardial infarction. In addition, future studies should focus in particular on the prognostic relevance regarding long-term outcomes, such as the development of heart failure or the occurrence of recurrences.

### Strengths and limitations

This study included patients from the Augsburg Myocardial Infarction Registry with consecutive enrollment, minimizing selection bias. Blood samples were collected during PCI intervention and processed in a standardized manner, ensuring high sample quality. Comprehensive characterization of STEMI cases allowed for adjustment for multiple confounders in the analyses. However, the study has several limitations. The findings could not be replicated in an independent cohort of STEMI patients. The results of this study apply to the amino acids investigated, so associations with other amino acids could not be assessed. Furthermore, the present study did not account for all comorbidities that can influence the concentrations of plasma metabolites and are common in patients with STEMI, such as metabolic syndrome. In addition, long-term sample storage as a potential source of variability could have influenced the present findings. As an observational study, causal relationships cannot be established. Amino acid levels are influenced by recent food intake as well as by the use of medications such as statins, beta-blockers, ACE inhibitors, dietary supplements, and corticosteroids. Since no data is available on these confounding factors, the possibility of residual or unmeasured confounding remains. Another limitation of the study is that amino acid concentrations were measured at only a single point in time. As a result, changes in concentration over time following the acute event could not be considered. Furthermore, this study examined only the association with 28-day mortality, so it is not possible to draw conclusions about long-term prognosis. Finally, since the study population consisted only of STEMI patients aged 25 to 84 years, the results may not be generalizable to other age groups, ethnicities, or to patients with non-ST-elevation myocardial infarction.

## Conclusions

The present findings suggest that the acute phase of STEMI is associated with changes in plasma amino acid levels that may reflect alterations in energy metabolism and metabolic stress.

By demonstrating that these amino acid fluctuations are associated with 28-day mortality, this research highlights the potential of metabolomic profiling to refine early risk stratification. Ultimately, understanding these acute metabolic disruptions may inform future research into strategies aimed at restoring metabolic homeostasis and improving short-term survival in patients with acute myocardial infarction.

## Data Availability

The data underlying this article cannot be shared publicly because the data are subject to national data protection laws and restrictions that were imposed by the ethics committee of the Bavarian Medical Association (“Bayerische Landesärztekammer”) to ensure data privacy of the study participants because they did not explicitly consent to the data being made publicly available. The data will be shared at reasonable requests to the corresponding author.
